# Complete Genome Sequence of a European Genotype Porcine Reproductive and Respiratory Syndrome Virus in China

**DOI:** 10.1128/genomeA.00175-13

**Published:** 2013-05-09

**Authors:** Zhi Zhou, Qi Liu, Dongmei Hu, Xiaojuan Yue, Xiuling Yu, Qian Zhang, Xiaoxue Gu, Jianqiang Ni, Xiangdong Li, Xinyan Zhai, Kegong Tian

**Affiliations:** Veterinary Diagnosis Center and OIE Porcine Reproductive and Respiratory Syndrome Laboratory, China Animal Disease Control Center, Chaoyang District, Beijing, People’s Republic of Chinaa;; Department of Anatomy and Physiology, College of Veterinary Medicine, Kansas State University, Manhattan, Kansas, USAb

## Abstract

Here, we report a novel European genotype porcine reproductive and respiratory syndrome virus (PRRSV) strain with 15 nucleotide deletions in the nonstructural protein 2 region and 3 nucleotide deletions in the overlapping regions of the open reading frame 3 (ORF3) and ORF4 regions. This study will aid in further exploration of the genetic and antigenic diversity of the European genotype of the PRRSV in China.

## GENOME ANNOUNCEMENT

Porcine reproductive and respiratory syndrome virus (PRRSV), a member of the *Arteriviridae* family in the order *Nidovirales*, is an enveloped, positive-sense, single-stranded RNA virus that causes reproductive failure in pregnant sows and respiratory distress in young pigs (1, 2). PRRS occurs in most pig-producing countries worldwide and causes enormous economic losses in the swine industry (3). A significant characteristic of PRRSV is the extensive genetic variation within the different isolates in the field (4). Based on genetic and antigenic differences, the PRRSVs have been divided into two major genotypes, the European genotype (EU) (type 1, prototype Lelystad virus) and the North American genotype (NA)( type 2, prototype VR-2332). The two genotypes share only about 60% identity on the nucleotide level (2, 5). In China, the North American genotype PRRSV and the European genotype PRRSV were first reported in 1996 and 2006 (6, 7), respectively. To further explore the genetic diversity of the European genotype PRRSV in China, here we report the complete genome sequence of the European genotype PRRSV NVDC-NM1-2011.

The NVDC-NM1-2011 PRRSV was isolated from naturally infected swine herds in 2011. The virus total RNA was extracted from sera of infected swine, and the complete genome of NVDC-NM1-2011 was generated with reverse transcriptase PCR (RT-PCR) using 16 pairs of primers amplifying 16 overlapped fragments of PRRSV (6). The 16 amplified RT-PCR products were purified and cloned into a pGEM-T Easy vector (Promega) and sequenced with an automated sequencer (Genetic analyzer 3730XL; Applied Biosystems). The complete genomic sequence of NVDC-NM2-2011 is 15,081 nucleotides (nt), excluding the 3′ poly(A) tail. Comparison of NVDC-NM1-2011 with the EU PRRSV prototypic strain Lelystad virus by whole-genome BLAST techniques showed that there are 18-nt discontinuous deletions, which are 15-nt deletions in the Nsp2 region (12-nt deletions at positions 2224 to 2235 and 3-nt deletions at positions 2374 to 2376) and 3-nt deletions within the 252-nt overlapping regions of the open reading frame 3 (ORF3) and ORF4 regions (at positions 202 to 204). Previous studies have indicated the nonstructural Nsp2, ORF3, and ORF5 sequences are most variable among the different strains within the genome of PRRSV, which are likely to be associated with the virulence of PRRSV (8–10). In the future, investigations should be undertaken to determine whether these mutations of the NVDC-NM1-2011 isolate will lead to pathogenic changes in swine. In addition, PRRSV shares 90.77% nucleotide identity with the genome of the prototype Lelystad virus.

In all, the sequence data of NVDC-NM1-2011 indicate that the EU genotype PRRSV variant prevails in China. This information will aid in further exploration of the genetic and antigenic diversity of the European genotype of PRRSV in China.

### Nucleotide sequence accession number.

The complete genome sequence of NVDC-NM1-2011 is available in GenBank under the accession number JX187609.

## References

[1] Benfield DA Nelson E Collins JE Harris L Goyal SM Robison D Christianson WT Morrison RB Gorcyca D Chladek D 1992 Characterization of swine infertility and respiratory syndrome (SIRS) virus (isolate ATCC VR-2332). J. Vet. Diagn. Invest. 4:127–133 1616976 10.1177/104063879200400202

[2] Meulenberg JJ Hulst MM de Meijer EJ Moonen PL den Besten A de Kluyver EP Wensvoort G Moormann RJ 1993 Lelystad virus, the causative agent of porcine epidemic abortion and respiratory syndrome (PEARS), is related to LDV and EAV. Virology 192:62–72 8517032 10.1006/viro.1993.1008 PMC7173055

[3] Neumann EJ Kliebenstein JB Johnson CD Mabry JW Bush EJ Seitzinger AH Green AL Zimmerman JJ 2005 Assessment of the economic impact of porcine reproductive and respiratory syndrome on swine production in the United States. J. Am. Vet. Med. Assoc. 227:385–392 16121604 10.2460/javma.2005.227.385

[4] Meng XJ 2000 Heterogenicity of porcine reproductive and respiratory syndrome virus: implications for current vaccine efficacy and future vaccine development. Vet. Microbiol. 74:309–329 10831854 10.1016/S0378-1135(00)00196-6 PMC7117501

[5] Nelsen CJ Murtaugh MP Faaberg KS 1999 Porcine reproductive and respiratory syndrome virus comparison: divergent evolution on two continents. J. Virol. 73:270–280 9847330 10.1128/jvi.73.1.270-280.1999 PMC103831

[6] Chen N Cao Z Yu X Deng X Zhao T Wang L Liu Q Li X Tian K 2011 Emergence of novel European genotype porcine reproductive and respiratory syndrome virus in mainland China. J. Gen. Virol. 92:880–892 21216986 10.1099/vir.0.027995-0

[7] Guo BQ Chen ZS Liu WX Cui YZ 1996 Isolation and identification of porcine reproductive and respiratory syndrome (PRRS) virus from aborted fetuses suspected of PRRS. Chin. J. Anim. Poult. Infect. Dis. 26:1–5

[8] Fang Y Kim DY Ropp S Steen P Christopher-Hennings J Nelson EA Rowland RR 2004 Heterogeneity in Nsp2 of European-like porcine reproductive and respiratory syndrome viruses isolated in the United States. Virus Res. 100:229–235 15019241 10.1016/j.virusres.2003.12.026

[9] Music N Gagnon CA 2010 The role of porcine reproductive and respiratory syndrome (PRRS) virus structural and non-structural proteins in virus pathogenesis. Anim. Health Res. Rev. 14:1–29 10.1017/S1466252310000034 20388230

[10] Ostrowski M Galeota JA Jar AM Platt KB Osorio FA Lopez OJ 2002 Identification of neutralizing and nonneutralizing epitopes in the porcine reproductive and respiratory syndrome virus GP5 ectodomain. J. Virol. 76:4241–4250 11932389 10.1128/JVI.76.9.4241-4250.2002 PMC155073

